# Jianpi Huayu Decoction Inhibits Proliferation in Human Colorectal Cancer Cells (SW480) by Inducing G0/G1-Phase Cell Cycle Arrest and Apoptosis

**DOI:** 10.1155/2015/236506

**Published:** 2015-09-17

**Authors:** Song-yang Xi, Yu-hao Teng, Yan Chen, Jie-ping Li, Ying-ying Zhang, Shen-lin Liu, Xi Zou, Jin-yong Zhou, Jian Wu, Rui-ping Wang

**Affiliations:** ^1^The Affiliated Hospital of Nanjing University of Chinese Medicine, Nanjing, Jiangsu 210029, China; ^2^Nanjing University of Chinese Medicine, Nanjing, Jiangsu 210023, China

## Abstract

Jianpi Huayu Decoction (JHD), a Chinese medicine formula, is a typical prescription against multiple tumors in the clinical treatment, which can raise quality of life and decrease complications. The aim of this study is to assess the efficacy of JHD against human colorectal carcinoma cells (SW480) and explore its mechanism. MTT assay showed that JHD decreased the cellular viability of SW480 cells in dose-dependent and time-dependent manner. Flow cytometry analysis revealed that JHD induced G0/G1-phase cell cycle arrest in SW480 cells and had a strong apoptosis-inducing effect on SW480 cells. Meanwhile it enhanced the expression of p27, cleaved PARP, cleaved caspase-3, and Bax and decreased the levels of PARP, caspase-3, Bcl-2, CDK2, CDK4, CDK6, cyclin D1, cyclin D2, cyclin D3, and cyclin E1, which was evidenced by RT-qPCR and Western blot analysis. In conclusion, these results indicated that JHD inhibited proliferation in SW480 cells by inducing G0/G1-phase cell cycle arrest and apoptosis, providing a practicaltherapeutic strategy against colorectal cancer.

## 1. Introduction

Colorectal cancer (CRC) is one of the most common malignant tumors, which is characterized by high morbidity and mortality [1]. At present, multiple chemotherapeutic drugs are widely used in the treatment of CRC, but their side effects hamper their clinical application and efficacy, such as neurotoxic effects and myelosuppression [2]. However, compared with chemotherapeutic drugs, natural products contain relatively fewer side effects and have been shown to possess beneficial therapeutic effects for cancer [3–5]. Therefore, it is necessary to develop naturally occurring agents against CRC.

According to the major precepts of traditional Chinese medicine (TCM), these Chinese formulas emphasize not only the symptoms but also restoring and maintaining the body homeostasis, which is very similar to the rationale of modern multitargeted therapeutics [6]. The Chinese herbal formula, Jianpi Huayu Decoction (JHD), contains* Atractylodes macrocephala *Koidz.,* Euphorbia humifusa *Willd.,* Salvia miltiorrhiza *Bunge.,* Paris polyphylla *Sm.,* Curcuma phaeocaulis *Val.,* Scutellaria barbata *D. Don., and* Artemisia capillaris *Thunb. and has been well used against CRC in the clinical treatment, which can raise quality of life and decrease complications [7]. Previous studies proposed that extracts of JHD possess antitumor activity to suppress the growth of many types of cancer including CRC both in vitro and in vivo [7, 8]. To further elucidate the precise mechanism of the potential tumoricidal activity of JHD, in this study, we investigated its antiproliferative activity against human colon adenocarcinoma SW480 cells and possible mechanisms of growth inhibition.

## 2. Materials and Methods

### 2.1. Chemicals and Antibodies

RPMI 1640 and calf serum were purchased from HyClone (Thermo Scientific, USA). 3-(4,5-Dimethylthiazol-2-yl)-2,5-diphenyltetrazolium bromide (MTT) and monoclonal mouse *β*-actin antibody were purchased from Sigma Chemical Co. (St. Louis, MO). The antibodies against Bax, Bcl-2, cyclin D1, cyclin D2, cyclin D3, cyclin E1, CDK2, CDK4, CDK6, p27, caspase-3, cleaved caspase-3, PARP, and cleaved PARP and the horseradish peroxidase (HRP) labeled goat anti-mouse or anti-rabbit IgG antibody were purchased from Cell Signaling Technology (Beverly, MA). Annexin V-fluorescein isothiocyanate (FITC)/PI apoptosis detection kit and Cell Cycle Analysis kit were from BD Biosciences (San Diego, CA). TRIzol reagent and Power SYBR Green PCR Master Mix were from Life Technologies (Grand Island, NY). PrimeScript RT reagent Kit with gDNA Eraser was from TaKaRa (Dalian, China). All other chemicals were purchased from Sigma and Merck and were of the highest grade available.

### 2.2. Preparation of the JHD Extract

The herbs of the JHD extract (*Atractylodes macrocephala *Koidz. 10 g,* Euphorbia humifusa *Willd. 10 g,* Salvia miltiorrhiza *Bunge. 15 g,* Paris polyphylla *Sm. 15 g,* Curcuma phaeocaulis *Val. 10 g,* Scutellaria barbata *D. Don 30 g, and* Artemisia capillaris *Thunb. 15 g) were obtained from the Jiangsu Province Hospital of Traditional Chinese Medicine (Nanjing, China). The total weight of the dried herbs was 105 g. First, the plants were blended in 1050 mL double-distilled water (1 : 10, w/v) for 1 h and then heated to 100°C for 2 h, after which the residue was boiled for 2 h with 1050 mL distilled water again. Next, the two extracts were mixed and concentrated to 1 g herb/mL and then filtered through a 0.2 *μ*m filter. The extract was stored at −20°C until use.

### 2.3. Cell Lines and Cell Culture

The human colorectal carcinoma SW480 cells were provided by the Center Laboratory of the Jiangsu Province Hospital of Traditional Chinese Medicine (Nanjing, China), cultured in RPMI-1640 medium containing 10% bovine serum, penicillin (100 U/mL), and streptomycin (100 *μ*g/mL) at 37°C in 5% CO_2_ atmosphere.

### 2.4. MTT Assay

The SW480 cells in the logarithmic growth phase were seeded on the 96-well plate at 8 × 10^3^ cells/well and incubated overnight. After the cells were treated with JHD (0, 0.25, 0.5, 1, 2, 4, and 8 mg/mL) for 12 h, 24 h, and 48 h, the medium was discarded and treated with 20 *μ*L MTT for 4 h at 37°C. Cells were lysed in 150 *μ*L DMSO, and then the optical densities (ODs) were measured at 490 nm by using ELx800 microplate reader (BioTek, Winooski, VT). The cell growth inhibition rate was calculated using the following formula: Inhibition rate = (1 − OD_experiment_/OD_control_) × 100%.

### 2.5. Apoptosis Assay

The SW480 cells (1 × 10^5^) were seeded into 60 mm Petri dish and incubated overnight. After treatment with or without JHD (1, 2, 4 mg/mL) for 24 h, SW480 cells were harvested, washed twice with cold phosphate buffer saline (PBS), and resuspended in 1x binding buffer, to which 5 *μ*L Annexin V-FITC and 5 *μ*L propidium iodide (PI) were added. The cells were gently vortexed and incubated for 15 min at room temperature in the dark and then analyzed by flow cytometry (FCM).

### 2.6. Cell Cycle Analysis

The SW480 cells (1 × 10^6^) were seeded into 100 mm Petri dish and incubated overnight. After synchronization by changing the media with a serum-free media for 24 h, the cells were then treated with or without JHD (1, 2, 4 mg/mL) for 24 h. Next, the cells were harvested, washed with cold PBS, and then fixed with 70% cold ethanol at 4°C overnight. After being washed twice with cold PBS, fixed cells were resuspended with 100 *μ*g/mL RNase, incubated with 50 *μ*g/mL PI at 37°C for 30 min in the dark room, and analyzed by FCM.

### 2.7. RT-qPCR Analysis

The SW480 cells (8 × 10^5^) were seeded into 60 mm Petri dish and incubated overnight. After treatment with or without JHD (1, 2, 4 mg/mL) for 24 h, the total RNA was isolated using TRIzol reagent and reverse-transcribed into cDNA using the TaKaRa RT reagent kit. The PCR reactions were quantified by ABI 7500 fast RT-qPCR System. The sequences of the primers were provided in Table 1. Each sample was tested in triplicate. Cycle threshold (Ct) values were obtained graphically for the target genes and GAPDH. ΔCt = Ct_target  genes_ − Ct_GAPDH_. ΔΔCt = ΔCt_treated  samples_ − ΔC_tcontrol  samples_. The relative fold change in gene expression was calculated as 2^−ΔΔCT^.

### 2.8. Western Blot Analysis

The SW480 cells (2 × 10^6^) were seeded into 100 mm Petri dish and incubated overnight. After treatment with or without JHD (1, 2, 4 mg/mL) for 24 h, SW480 cells were washed twice with cold PBS, lysed in RIPA buffer containing protease inhibitor cocktail (P8340, Sigma-Aldrich) for 30 min on ice, and then harvested by using cell scraper. Cell lysates were centrifuged at 12000 g for 15 min at 4°C. Protein concentrations were determined by the Bradford method. Samples containing equal proteins (20 *μ*g) were loaded and analyzed by Western blot assay. Briefly, proteins were separated by 10% sodium dodecyl sulfate polyacrylamide gel electrophoresis (SDS-PAGE) and transferred onto polyvinylidene fluoride (PVDF) membrane (Millipore, Billerica, MA). Membrane were washed by Tris-buffered saline Tween (TBST, 20 mM Tris-HCl, pH 7.6, 150 mM NaCl, and 0.01% Tween-20) for 5 min and incubated with blocking buffer (TBST containing 50 g/L BSA) for at least 1 h at room temperature. Membranes were incubated with desired primary antibody Bax, Bcl-2, cyclin D1, cyclin D2, cyclin D3, cyclin E1, CDK2, CDK4, CDK6, p27, caspase-3, cleaved caspase-3, PARP, cleaved PARP (at a dilution of 1 : 1,000), and *β*-actin (at a dilution of 1 : 5,000) overnight at 4°C. Then, membranes were washed in TBST for 5 min three times and incubated with HRP-linked secondary antibodies (anti-rabbit or anti-mouse IgG, at a dilution of 1 : 1,000) for 1 h at room temperature. After the membranes were washed for 5 min three times with TBST, the signals were detected by an ECL detection kit (Millipore, Billerica, MA). *β*-actin was used as a loading control.

### 2.9. Statistical Analysis

All data were evaluated using analysis of one-way ANOVA test with SPSS 19.0 which were expressed as the mean ± standard deviation (SD). Significant differences between the control and treated cells were defined as *P* < 0.05 or 0.01.

## 3. Results

### 3.1. JHD Suppressed SW480 Cells Proliferation In Vitro

**Figure 1:**
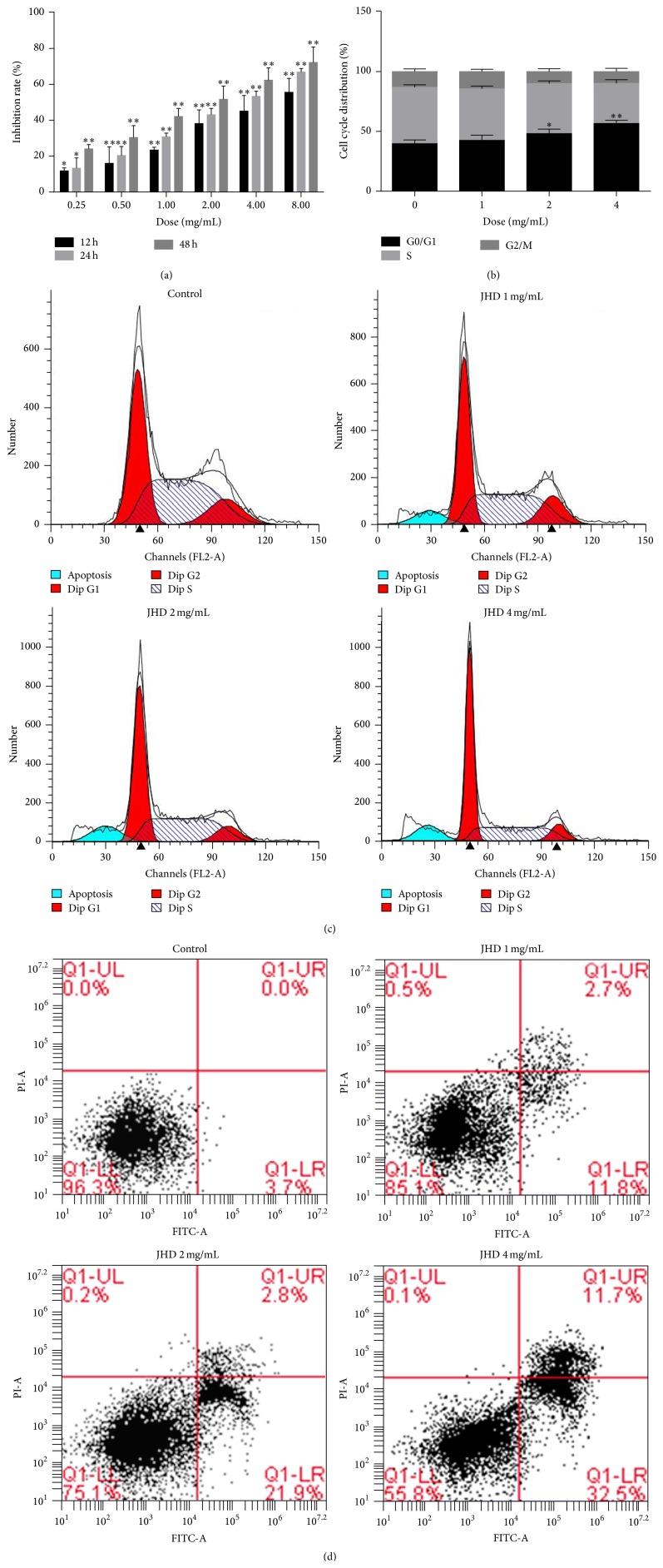
(a) The effect of JHD on the viability of SW480 cells. Cells were treated with different concentrations of JHD and for 12 h, 24 h, or 48 h. The bars indicate standard errors. The asterisk indicates a significant increase in the inhibition rate between groups treated with JHD and the untreated control group. Data are expressed as the mean ± SD of three experiments (^*∗*^ *P* < 0.05, ^*∗∗*^ *P* < 0.01). (b) and (c) The effect of JHD on cell cycle in SW480 cells. SW480 cells were treated with different concentration of JHD for 24 h. At the end of treatment, cells were trypsinized, incubated with RNase, stained with propidium iodide (PI), and analyzed by FCM. Data are expressed as the mean ± SD of three experiments. ^*∗*^ *P* < 0.05, ^*∗∗*^ *P* < 0.01, versus control cells. (d) The apoptosis induced by JHD in SW480 cells. Cultures of SW480 cells were treated with different concentration of JHD for 24 h harvested by trypsinization and centrifugation and then analyzed by flow cytometry after staining with annexin V-FITC and propidium iodide. Results shown are of an experiment representative of apoptosis. Q1-UL showed that cells were undergoing necrosis, and Q1-UR showed that cells were at the end stage of apoptosis. Q1-LL showed that cells were viable, or there was no measurable apoptosis. Q1-LR showed that cells were undergoing apoptosis.

We first examined the effect of JHD on SW480 cell viability by MTT assay. As shown in Figure 1(a), after treatment with JHD (0, 0.25, 0.5, 1, 2, 4, and 8 mg/mL) for 12 h, 24 h, and 48 h, the inhibition rates of the cells increased significantly compared to untreated control cells (*P* < 0.01 or 0.05). These results suggested that JHD inhibited the proliferation of SW480 cells in dose-dependent and time-dependent manner.

### 3.2. JHD Induced SW480 Cells Apoptosis

To further confirm the apoptosis of JHD, SW480 cells were stained with Annexin V/PI and subsequently analyzed by FCM after treatment with or without JHD (1, 2, and 4 mg/mL) for 24 h. As shown in Figure 1(d), from 0 to 4 mg/mL, early apoptosis rates of SW480 cells increased from 3.7 ± 0.56% to 32.5 ± 2.12%. In summary, the above results suggested that JHD could effectively induce apoptosis, and the apoptosis rates presented a dose-dependent manner.

### 3.3. JHD Induced G0/G1-Phase Cell Cycle Arrest in SW480 Cells

To further confirm the effect of JHD on cell cycle, SW480 cells were evaluated by FACS analysis with PI staining after treatment with or without JHD (1, 2, 4 mg/mL) for 24 h. As shown in Figures 1(b) and 1(c), from 0 to 4 mg/mL, the percentage of SW480 cells in G0/G1-phase increased from 39.76 ± 3.21% to 56.74 ± 2.62%, suggesting that JHD induced G0/G1-phase cell cycle arrest in SW480 cells in a dose-dependent manner.

### 3.4. JHD Altered the Expression of Cell Cycle Regulatory and Apoptotic Factors in SW480 Cells

**Figure 2:**
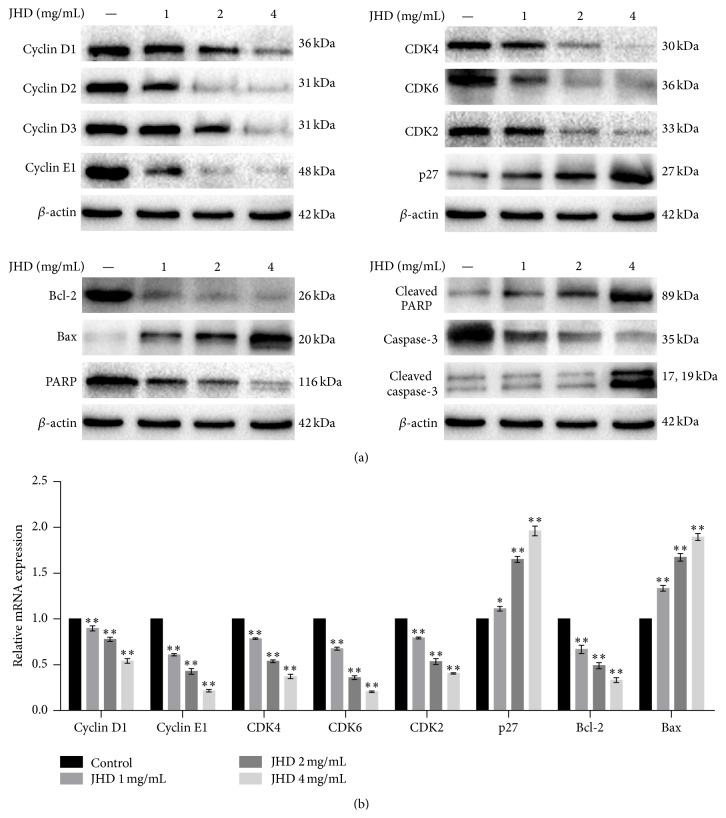
The effect of JHD on the expression of the cell cycle and apoptosis associated molecules in SW480 cells. Cells were treated with various concentrations of JHD for 24 h. (a) The protein levels of cyclin D1, cyclin D2, cyclin D3, cyclin E1, CDK2, CDK4, CDK6, p27, Bax, Bcl-2, PARP, cleaved PARP, caspase-3, and cleaved caspase-3 were determined by Western blotting. (b) The mRNA levels of cyclin D1, cyclin E1, CDK2, CDK4, CDK6, p27, Bax, and Bcl-2 were determined by RT-PCR. ^*∗*^ *P* < 0.05, ^*∗∗*^ *P* < 0.01, versus control cells. GAPDH and *β*-actin were used as the internal controls for the RT-PCR or Western blot assays.

To evaluate levels of cell cycle regulatory proteins* and apoptotic factors*, the expression levels were analyzed by RT-qPCR and Western blot analysis. As shown in Figure 2, JHD treatment significantly enhanced the expression levels of p27, Bax, cleaved caspase-3, and cleaved PARP and suppressed the expression levels of Bcl-2, cyclin D1, cyclin D2, cyclin D3, cyclin E1, CDK2, CDK4, CDK6, PARP, and caspase-3 in SW480 cells in a dose-dependent manner.

## 4. Discussion

TCM has been used in China to treat a variety of diseases including cancer. At present, TCM has gained increasing attention on its usage as an antitumor treatment, which is considered to be a multitarget agent that exerts therapeutic function in a holistic way. JHD has been well used against CRC in the clinical treatment, which can raise quality of life and decrease complications. To further elucidate the antitumor mechanism of JHD, herein we investigated its effect on the proliferation of human colon carcinoma SW480 cells in vitro.

Here we found that JHD inhibited proliferation of SW480 cells in dose-dependent and time-dependent manner. And JHD effectively induced apoptosis and G0/G1-phase cell cycle arrest, which presented a dose-dependent manner. Furthermore, RT-qPCR and Western blot analysis showed that JHD treatment significantly enhanced the expression levels of p27, Bax, cleaved caspase-3, and cleaved PARP and suppressed the expression levels of Bcl-2, cyclin D1, cyclin D2, cyclin D3, cyclin E1, CDK2, CDK4, CDK6, PARP, and caspase-3 in SW480 cells in a dose-dependent manner.

Numerous reports suggest that there is a strong link between cell cycle deregulation and carcinogenesis [9]. And deregulated growth is a unique characteristic of cancer cells and a primary requirement for processes in cancer progression [10]. Cellular proliferation is regulated primarily by the regulation of cell cycle to monitor DNA integrity [11], which consists of four distinct sequential phases (G0/G1, S, G2, and M) [12]. In eukaryotes, the cell cycle is regulated by cyclins, cyclin-dependent kinases (CDKs), and cyclin-dependent kinase inhibitors (CDKIs) [13]. The levels of CDKs remain constant during the cell cycle, whereas the levels of cyclins fluctuate, and the phases of the cell cycle are controlled by the activation of different CDK/cyclin complexes [14], the importance of which in cell proliferation is supported by the finding that deregulation of CDK/cyclin complex activity is observed in a variety of human tumors [15]. At different phases, passage through the cell cycle is governed by sequential activation and subsequent inactivation of a series of CDKs, whose activity depends on interactions with timely expressed cyclins and CDKIs [16]. Cell cycle arrest in response to stress is integral to the maintenance of genomic integrity, which occurs due to the loss of cyclin expression and CDK activity [17]. The control mechanisms that restrain cell cycle transition or induce apoptotic signaling pathways after cell stress are known as cell cycle checkpoints [18]. By using FCM analysis we found that the inhibitory effect of JHD on SW480 cell proliferation was associated with the blockage of G1 to S progression. As one of the main checkpoints of cell cycle, G1/S transition is responsible for initiation and completion of DNA replication, which is strongly regulated by the combined activity of the cyclin D/CDK4, cyclin D/CDK6, and cyclin E/CDK2 complex [19–21]. The proliferation inhibitor p27 plays an inhibitory role in G1/S progression by inhibiting the activity of cyclin/CDK complexes [22]. As shown in Figure 2, JHD upregulated the expression of p27 and downregulated the expression of cyclin D1, cyclin D2, cyclin D3, cyclin E1, CDK4, CDK6, and CDK2 in SW480 cells.

Apoptosis is a process of programmed cell death, which plays a crucial role in maintaining cellular homeostasis between cell division and cell death [23]. Caspase-cascade is a central part of cell apoptosis and regulated by various kinds of molecules, such as Bcl-2 family proteins. Generally, the caspase family proteases can be activated through two pathways: one is the death signal-induced, death receptor mediated pathway; the other is the mitochondrion-dependent pathway [24, 25]. Caspase-3 is the key enzyme required in the caspase cascade activation and execution [26]. Once the specificity substrate such as PARP has been cut by the cleavage of caspases, apoptosis will be induced [27, 28]. The present research used FCM to detect cell apoptosis rate, finding the apoptosis rates of SW480 cells being increased significantly when treated with JHD for 24 h. Further Western blot data revealed that the caspase-3, cleaved caspase-3, PARP, and cleaved PARP were activated by JHD, which indicated the cell apoptosis. Therefore, we inferred that activating caspase cascade is the mechanism of JHD inducing cell apoptosis. However, more research is in need to investigate JHD inducing the apoptosis of SW480 cells through endogenous pathways or exogenous pathways or through both. Although the caspase-cascade is a central point to apoptosis, its activation is regulated by many other factors, among which Bcl-2 family plays a pivotal role in either inhibiting (Bcl-2) or promoting (Bax) cell death [29, 30]. Recently, it has been reported that Bax inactivating Bcl-2 proteins regulates the apoptosis mediated by mitochondria and the ratio of Bax to Bcl-2 proteins increases during apoptosis induction [31]. Therefore, we continued to detect the expression change of Bcl-2 family. As shown in Figure 2, the protein level of Bax increased in a dose-dependent manner, while the Bcl-2 protein decreased.

## 5. Conclusion

According to the above results, we found that JHD could inhibit proliferation of SW480 cells by inducing G0/G1-phase cell cycle arrest and apoptosis. Its induction of apoptosis may activate the caspase-cascades and upregulate the expression of Bax and downregulate the expression of Bcl-2. JHD could induce G0/G1-phase cell cycle arrest of SW480 cells. Its molecular mechanism may lie on upregulating the expression of p27 and downregulating the expression of cyclin D1, cyclin D2, cyclin D3, cyclin E1, CDK4, CDK6, and CDK2. These findings provide an experiment basis for JHD as chemotherapy drugs against CRC cells, thereby facilitating the development of new anticancer agents.

## Figures and Tables

**Table 1:** Sequences of primers used in the real-time qPCR amplifications.

Gene	Primer sequences (5′-3′)	Length of PCR product (bp)
Cyclin D1	F: ACCTGAGGAGCCCCAACAAC	112
	R: GCTTCGATCTGCTCCTGGC	

Cyclin E1	F: GTCCTGGATGTTGACTGCCTTGA	258
	R: GTCCAGCAAATCCAAGCTGTCTC	

CDK2	F: GTCCAGCAAATCCAAGCTGTCTC	237
	R: CTGCTCTCACTGGCATTCCT	

CDK4	F: CTCTCTAGCTTGCGGCCTG	209
	R: GGCACCGACACCAATTTCAG	

CDK6	F: CTGCAGGGAAAGAAAAGTGC	95
	R: CTCCTCGAAGCGAAGTCCTC	

p27	F: TGCAACCGACGATTCTTCTACTCAA	185
	R: CAAGCAGTGATGTATCTGATAAACAAGGA	

Bax	F: TTTGCTTCAGGGTTTCATCC	213
	R: GCCACTCGGAAAAAGACCTC	

Bcl-2	F: TCGCCCTGTGGATGACTGAG	143
	R: CAGAGTCTTCAGAGACAGCCAGGA	

GAPDH	F: AGCCACATCGCTCAGACAC	66
	R: GCCCAATACGACCAAATCC	
